# Investigating cardiovascular risk in premenopausal women on oral contraceptives: Systematic review with meta-analysis

**DOI:** 10.3389/fcvm.2023.1127104

**Published:** 2023-04-25

**Authors:** Oyesanmi A. Fabunmi, Phiwayinkosi V. Dludla, Bongani B. Nkambule

**Affiliations:** ^1^ School of Laboratory Medicine and Medical Sciences (SLMMS), College of Health Sciences, University of KwaZulu-Natal, Durban, South Africa; ^2^ Department of Physiology, Ekiti State University, Ado-Ekiti, Nigeria; ^3^ Biomedical Research and Innovation Platform, South African Medical Research Council, Tygerberg, South Africa

**Keywords:** oral contraceptives, combined oral contraceptives, ethinylestradiol, progestins, cardiovascular disease

## Abstract

**Background:**

The use of oral contraceptives (OCs) is associated with an increased risk of cardiovascular events such as arterial and venous thrombosis (VTE). Cardiovascular diseases (CVDs) are the leading cause of death worldwide, with low- and middle-income nations accounting for over three-quarter of CVD deaths. The aim of this systematic review is to provide a comprehensive synthesis of the available evidence on the link between OC use and CVD risk in premenopausal women and to further assess the role of geographic disparities in the reported prevalence of CVD risk in women on OCs.

**Methods:**

A comprehensive search of databases such as MEDLINE, Academic Search Complete, Cumulative Index to Nursing and Allied Health Literature (CINAHL), and Health Source: Nursing/Academic Edition was conducted, right from the inception to the present, by using the EBSCOhost search engine. The Cochrane Central Register of Clinical trials (CENTRAL) was also searched to augment relevant sources of information. OpenGrey, which is a repository of information providing open access to bibliographical references, was searched and the reference list of the selected studies was also scanned. The potential risk of bias of the included studies was assessed using the modified Downs and Black checklist. Data analysis was performed using the Review Manager (RevMan) version 5.3.

**Results:**

We included 25 studies that comprised 3,245 participants, of which 1,605 (49.5%) are OC users, while 1,640 (50.5%) are non-OC users. A total of 15 studies were included for meta-analysis, and the overall pooled estimates suggested a significant increase in the traditional cardiovascular risk variables [standardized mean difference (SMD) = 0.73, (0.46, 0.99) (*Z* = 5.41, *p* < 0.001)] and little to no difference in endothelial activation among OC users when compared with non-OC users [SMD = −0.11, (−0.81, 0.60) (*Z* = 0.30, *p* = 0.76)]. Europe [SMD = 0.03, (−0.21, 0.27), (*Z* = 0.25 *p* = 0.88)] had the least effect size, while North America had the highest effect size [SMD = 1.86, (−0.31, 4.04), (*Z* = 1.68 *p* = 0.09)] for CVD risk in OC users when compared with non-OC users.

**Conclusion:**

The use of OCs suggests a significant increase in the prevalence of traditional cardiovascular risk variables with little to no difference in the risk of endothelial dysfunction when compared with non-OC users, and the magnitude of CVD risks varies across different geographical regions.

**Registration and protocol:**

This systematic review was registered in the international prospective register of systematic reviews (PROSPERO) under the registration number: CRD42020216169.

## 1. Introduction

Hormonal contraceptives, primarily oral contraceptive pills (OCPs), are one of the most commonly-prescribed modern methods of birth control for premenopausal women aged 15–49 (1) because of its high efficacy and safety profile (2–5). There are an estimated 151 million women using OCPs worldwide (6) and developed countries account for over 30% of such women (6, 7). Despite the known health benefits of OCPs that include preventing pregnancy and treating reproductive disorders among others (8–10), their physiological impact on women's health, combined with the risk of cardiovascular events (2, 11) such as arterial and venous thrombosis (ATE and VTE), ischemic and hemorrhagic stroke, and myocardial infarction (12–15) at various phases of life, remains a major concern (1). Nonetheless, a previous report showed that the incidence of cardiovascular events is rare in young female adults (1–2 per 10,000 per year) but the rate increases to ∼1% per year in the elderly (16, 17), indicating age as a strong predisposing risk factor of cardiovascular disease (CVD) among women, especially in developed countries (18, 19).

Since the introduction of the first-generation combined oral contraceptives (COCs), efforts to reduce their adverse cardiovascular side effects has led to the development of subsequent second-generation and third-generation medications (levonorgestrel; LNG and desogestrel; DSG or norgestimate, respectively) with lower estrogen dose and a varying progestin component called “gonanes,” including the recent fourth-generation medication (drospirenone; DRSP) (1). However, emerging evidence shows conflicting differences regarding the individual impact of COCs on several cardiovascular risk variables such as metabolic, hemodynamic, and hemostatic parameters (1, 10, 13, 20–23), and their impact is attributed to the dose of estrogen and progestin type (24, 25) and the duration of use (26).

Notably, evidence from previous studies showed an association between third-generation COCs (desogestrel; DSG and gestodene; GSD) and elevated risk of thrombosis when compared with the second-generation COC (levonorgestrel) (27–29). More so, the reported incidence of thrombotic events associated with third-generation COCs, when compared with second- and fourth-generation COCs, remains high at 6.6 per 10,000-woman (27). However, the incidence rates for ATE events is lower in women on DRSP-containing OCs compared to other COCs (30, 31). More so, the relative risk of ATE for COCs containing 30–35 µg ethinylestradiol and gestodene, desogestrel, cyproterone acetate, or DRSP was similar, and approximately 50%–80% higher than, the second-generation LNG (32, 33).

In contrast, a previous study showed that the use of COCs are not associated with the occurrence of acute myocardial infarction in young women because no excess risk was reported among users of desogestrel and gestodene when compared with LNG (14). In fact, the study further reported a high amelioration of CVD-risk among smokers using the third-generation COC when compared with the second-generation LNG (14), which contradicts with the finding of another multicenter, case-control study that reported a 3-fold increased risk of ischemic stroke among COC users (34, 35). However, the incidence and risk of ischemic stroke attributable to OC use in the study was reportedly low in women of reproductive age who are non-smokers with no hypertension (34, 35).

Furthermore, a recent study showed an increased number of adverse events relating to CVD in fourth-generation COC (DRSP) users when compared with second/third-generation COC users, and the number of reported events was the highest in the 20-year age group, followed by the 30-year age group, and finally in those over 40 years (36). Meanwhile, available data on the risk of cardiovascular events among different formulations of COC remain inconclusive and further research is needed to identify the causality between COCs and CVDs (36). Therefore, the aim of this systematic review and meta-analysis was to provide a comprehensive synthesis of the available evidence on the link between COC use and CVD risk in premenopausal women and to further assess the role of geographic disparities in the reported prevalence of CVD risk in women on COCs.

## 2. Methods

This systematic review and meta-analysis was prepared according to the preferred reporting items for systematic reviews and meta-analysis (PRISMA) guidelines (37) and the protocol was published (38). A comprehensive and systematic search of published studies was conducted to address the following research questions:

1. Do COCs impact cellular and vascular markers of endothelial activation?
2. What is the role of COCs in traditional cardiovascular risk variables?

### 2.1. Eligibility criteria

We included cross-sectional, cohort, and case control studies and randomized control trials. Studies reporting on the effect of OC use as a method of contraception on the risk of CVDs in healthy premenopausal women were also included. There were no language restrictions.

### 2.2. Exclusion criteria

Reviews, books, letters to editors including gray literature were excluded, the bibliographies that were searched for relevant citations.

### 2.3. Search strategy and information sources

The search strategy was developed using medical subheadings (MeSHs) and keywords related to oral contraceptives, cardiovascular disease, and premenopausal women (Supplementary File S1). The keywords and MeSH terms used included oral contraceptive pills, premenopausal women, cardiovascular disease, or coronary heart disease. A comprehensive search of databases such as MEDLINE, Academic Search Complete, Cumulative Index to Nursing and Allied Health Literature (CINAHL), Health Source: Nursing/Academic Edition, APA PsycInfo, and MasterFILE Premier was conducted from inception to the present by using the EBSCOhost search engine. Furthermore, the Cochrane Central Register of Clinical trials (CENTRAL) was searched including OpenGrey (System Information on Grey Literature in Europe) (www.open.eu) to obtain relevant sources of information. In addition, the reference list of the selected studies was scanned, and forward citation tracking was done using Google scholar to identify the relevant literature. In instances of disagreements, a third reviewer (BBN) was consulted to conduct arbitration proceedings.

### 2.4. Study selection

The screening of studies was performed by two independent reviewers (OAF and PVD) to avoid inconsistencies with regard to the eligibility of the studies. The abstracts were screened, and the full texts of eligible studies were retrieved. In instances of discrepancies, BBN was consulted for arbitration.

### 2.5. Outcomes

The primary outcomes of this systematic review and meta-analysis were endothelial activation measured by nitric oxide (NO) and endothelin 1 (ET-1) level, flow-mediated dilation (FMD), and common carotid artery intima–media thickness (CCA-IMT). The secondary outcomes was cardiovascular risk evaluated by changes in blood pressure, lipid profile, and blood glucose levels.

### 2.6. Data items and collection process

A data extraction sheet was used to extract data items that included the name of the author, year of publication, country, population (sample size), study design, types of OC, dosage, and main findings of the study. Mendeley desktop reference manager software (version 1.19.4) was used to examine the retrieved citations and to remove study duplicates.

### 2.7. Quality assessment and risk of bias

The potential risk of bias of the included studies was assessed using the modified Downs and Black checklist (39). The tool assesses four domains, namely, reporting bias, external validity, internal validity, and selection bias. Each study was graded and scored as either “excellent” (24–28 points), “good” (19–23 points), “fair” (14–18 points), or “poor” (<14 points).

### 2.8. Certainty of evidence

The quality of evidence was evaluated using the grading of recommendations assessment, development, and evaluation (GRADE) tool (40). The findings are summarized and presented in the summary of findings table (Table 4).

### 2.9. Data synthesis and statistical analysis

Higgin's *I*^2^ statistic was used to assess statistical heterogeneity. In instances of substantial heterogeneity (*I*^2^ > 50%), a random-effects model was used to generate pooled effect estimates (41). Outcomes with same-effect estimates were reported as the mean difference (MD), while different-effect estimates were reported as the standardized mean difference (SMD) and a 95% confidence interval (CI). To explore potential sources of statistical heterogeneity, we conducted a subgroup analysis on the basis of the study design. Data analysis was performed using the software Review Manager (RevMan) version 5.3. The levels of inter-rater agreement were assessed using Cohen's kappa (39), in which a score of values 0.01–0.20 indicate none to slight agreement, 0.21–0.40 fair, 0.41–0.60 moderate, 0.61–0.80 substantial, and 0.81–1.00 an almost perfect agreement (42). A *p*-value of ≤0.05 was considered statistically significant.

### 2.10. Sensitivity analysis and publication bias

Sensitivity analysis was performed to test the robustness of our reported effect estimates by following a stepwise removal of studies. We performed repeated meta-analysis by taking into account participants’ characteristics and study design, and thereafter, sensitivity analysis was conducted on the basis of geographical location. Furthermore, the method of visual inspection of funnel plots was used to assess publication bias.

## 3. Results

**Figure 1:**
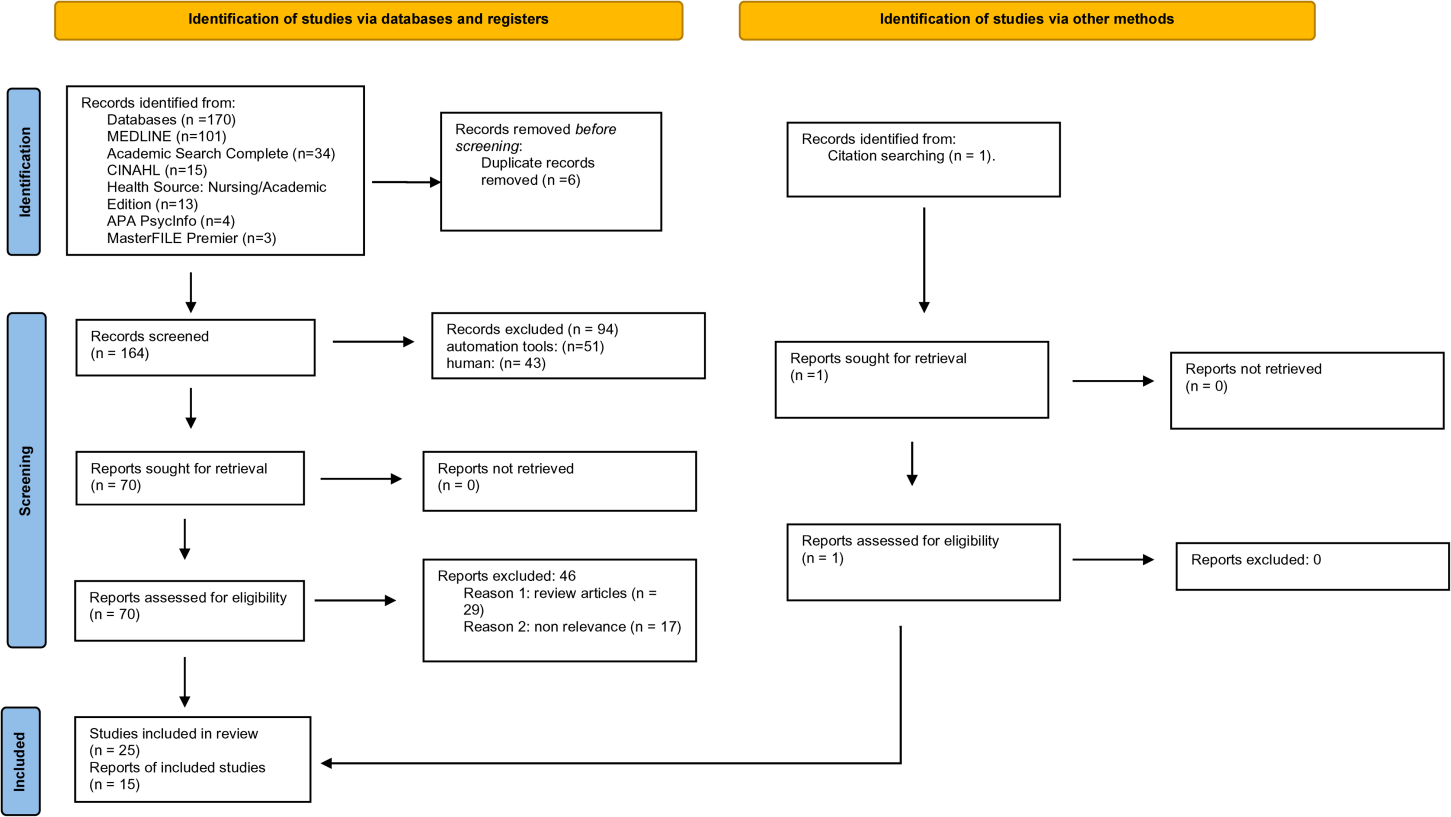
PRISMA flow diagram illustrating the study selection procedure.

A total of 165 studies were identified and retrieved using the search strategy and screened for eligibility. A total of 25 studies met the inclusion criteria, while total of 140 studies were excluded. Among the excluded studies, 17 were reviews, and 123 were not relevant to the topic of interest (Figure 1). In all, only 15 studies were shortlisted for quantitative and meta-analyses.

### 3.1. Characteristics of the included studies

The included studies were published between 1998 and 2019, and the study characteristics are given in Table 1. The included studies comprised 3,245 participants, of which 1,605 (49.5%) were on OCs, while 1,640 (50.5%) were non-OC users. Furthermore, 11 studies were cross-sectional studies (43–45, 47–50, 55, 56, 63, 64), seven were randomized control trials (51, 52, 59–61, 65, 66), three were cohort studies (54, 57, 62), two were clinical trials (53, 67), and one each was a prospective longitudinal study (46), and a case control study (58). In addition, the geographical distribution of the included studies comprised Europe (*n *= 6) (46, 51, 52, 58, 65, 66), North America (*n *= 6) (43–45, 47, 54, 57), South America (*n *= 6) (49, 53, 56, 59, 61, 67), Asia (*n *= 4) (50, 55, 62, 64), Africa (*n *= 2) (48, 63), and Australia (*n *= 1) (60).

**Table 1:** Characteristics of the included studies (*n* = 25).

Author	Year	Country	Main finding	Aim of study	Study design	Type of contraceptive	Dosage	Duration of use	Sample size	Age (years)
Friedman et al. (43)	2011	United States	OCPs significantly decreased serum iron and transferrin saturation and significantly increased FMD in the brachial artery.	To characterize the link between OCP use, iron stores, and cardiovascular risk in premenopausal women	Cross-sectional	COC	Estranes/gonanes and DRSP; 20–25 μg, >25 μg	>1 year	**23** (OC: 23; Non-user: NR)	OC (24.4 ± 2.3) Non-user (NR)
Blackmore et al. (44)	2011	Canada	OC use significantly lowered IGF-1 levels among younger women (18–21) when compared with non-users and older women (31–40).	Effect of past OC use and timing on circulating IGF-1	Cross-sectional	COC	NR	>1 year	**329** (OC: 137; Non-user:192)	OC (27.5 ± 2.1) Non-user (27.5 ± 2.1)
Meendering et al. (45)	2008	United States	MPA treatment antagonized estradiol administration by decreasing endothelium-dependent vasodilation and increasing resting plasma ET-1 in healthy young women.	To investigate whether medroxyprogesterone acetate (MPA) antagonizes the favorable effects of exogenous estradiol on vascular function and biomarkers of cardiovascular risk in young women.	Cross-sectional	Progestin only	5 mg MPA	10 days	**14** (OC: 14; Non-user: NR)	OC (23 ± 5.7) Non-user (NR)
Merki-Feld et al. (46)	2002	Switzerland	No significant changes in the plasma levels of nitric oxide, ET-1, blood pressure (BP), and BMI. However, there was a significant negative correlation between nitric oxide and endothelin-1, and nitric oxide and cholesterol, and a positive correlation between endothelin-1 and cholesterol.	To examine the influence of LNG and GSD on plasma levels of the vasodilator NO, the vasoconstrictor endothelin 1, and the plasma lipids, cholesterol and HDL	Prospective Longitudinal	COC	30 µg EE/150 µg LNG; 30 µg EE/75 µg GSD.	6 months	**12** (OC: 12; Non-user: NR)	OC (21.7 ± 4.3) Non-user (NR)
Odutayo et al. (47)	2015	Canada	Baseline levels of angiotensinogen, angiotensin II, aldosterone, and plasma renin activity were significantly higher in OCP subjects compared with normotensive control and contraceptive patch subjects, while the mean arterial pressure and BMI were non-significant.	Effects of the contraceptive patch and OCP on circulating renin–angiotensin–aldosterone system (RAAS) mediators and systemic blood pressure.	Cross-sectional study	COC, Transdermal patch	30 µg EE/150 µg LNG, 20 µg EE/150 µg norelgestromin	3 months	**25** (OC: 10; Non-user: 15)	OC (28 ± 1) Non-user (24 ± 1)
Asare et al. (48)	2014	Ghana	Diastolic blood pressure, TC, LDL, Castelli risk indices I (TC/HDL) and II (LDL/HDL), and BMI were significantly higher in the HC group than in the control group.	Effect of hormonal contraceptives on lipid profile and the risk indices for CVD in a Ghanaian community	Cross-sectional	COC, Progestin only	0.03 mg EE/0.15 mg LNG 0.035 mg norethindrone	>1 year	**43** (OC: 19; Non-user: 24)	OC (33.1 ± 6.3) Non-user (29.3 ± 8.1)
Lizarelli et al. (49)	2009	Brazil	The OC group had significantly lower FMD and HDL when compared with the control group, while the impact on CCA-IMT was not significantly different.	Both a combined oral contraceptive and depot medroxyprogesterone acetate impair endothelial function in young women.	Cross-sectional	COC, Progestin only	EE30 µg/LNG 150 µg DMPA (150 mg)	6 months	**75** (OC: 25; Non-user: 50)	OC (23.7 ± 3.2) Non-user (23.4 ± 3.6)
Heidarzadeh et al. (50)	2014	Iran	COC use significantly reduced FMD% in comparison with the control group, while the mean CCA-IMT was significantly but slightly higher when compared with the age-matched control group	The effect of low-dose combined oral contraceptive pills on brachial artery endothelial function and CCA-IMT thickness	Cross-sectional	COC	EE 30 μg/LNG150 μg	>1 year	**60**(OC: 30; Non-users: 30)	OC (33.3 ± 4.6) Non-users (33.9 ± 5)
Yildizhan et al. (51)	2009	Turkey	OCs resulted in significant reductions in systolic and diastolic BP and LDL levels and a significant increase in TG and HDL levels, resulting in increasing the HDL/LDL ratio.	Effects of two combined oral contraceptives containing ethinyl estradiol 30 μg combined with either gestodene or drospirenone on hemostatic parameters, lipid profiles, and BP	RCT	COC	EE 0.03 mg/GSD 0.075 mg, EE 0.03 mg/DRSP 3 mg	1 year	**160** (OC: 160; Non-user: NR)	OC (27.5 ± 10.6) Non-user (NR)
Wiegratz et al. (52)	2004	Germany	OCs caused significant changes in the hemostatic parameters by increasing the levels of fibrinogen, prothrombin fragment 1 + 2, factor VII, protein C, plasminogen, Plasmin-Alpha-2-Antiplasmin (PAP) complexes, and D-dimer, while total and free protein S, t-PA, and PAI levels were significantly reduced.	Effect of four oral contraceptives on hemostatic parameters	RCT	COC	30 µg EE/2 mg DNG, 20 µg EE/2 mg DNG, 10 µg EE/2 mg EV/2 mg DNG, 20 µg EE/100 µg LNG	6 months	**100** (OC: 100; Non-user: NR)	OC (26.5 ± 12) Non-user (NR)
Giribela et al. (53)	2012	Brazil	The contraceptive formulation did not cause any significant changes in BP, heart rate (HR), cardiac output (CO), total peripheral resistance (TPR), and arterial endothelial function.	Effect of combined oral contraceptives containing drospirenone on endothelial function and hemodynamic parameters in healthy young women	Clinical trial	COC	20 µg EE/3 mg DRSP	6 months	**71** (OC:43; Non-user: 28)	OC (29.2 ± 6.8) Non-user (30.6 ± 6.8)
Kharbanda et al. (54)	2014	United States	COCs were not associated with clinically meaningful changes in weight or blood pressure.	Initiation of oral contraceptives and changes in blood pressure and BMI in healthy adolescents	Observational cohort	COC	30/35 µg EE	>1 year	**1,422** (OC: 510; Non-user: 912)	OC (16.4 ± 1) Non-user (16.4 ± 1)
Fallah et al. (55)	2012	Iran	OCs significantly elevated the levels of homocysteine (HCY) and reduced the levels of NO concentration in the plasma.	Influence of oral contraceptive pills on homocysteine and nitric oxide levels	Cross-sectional	COC	30 µg EE/I50 µg LNG	>1 year	**100** (OC: 50; Non-user: 50)	OC (27.5 ± 10.6) Non-user (27.5 ± 10.6)
Dos Santos et al. (56)	2018	Brazil	There was a significant increase in the levels of TG, HDL-cholesterol, CRP, and systolic blood pressure values in COCs. There was also a significant increase in plasma-oxidized LDL values when compared with the control group.	Elevation of oxidized lipoprotein of low density in users of combined oral contraceptives	Cross-sectional	COC	150 µg LNG/30 µg EE	>1 year	**42** (OC: 21; Non-user: 21)	OC (23 ± 3.1) Non-user (23 ± 3.4)
Harvey et al. (57)	2015	United States	Blood pressure was significantly higher in OC users than in OC non-users. Muscle sympathetic nerve activity (MSNA) at rest, as well as CO and TPR, is similar between the two study groups.	Effect of oral contraceptive use on MSNA, and systemic hemodynamics in young women	Retrospective cohort study	COC	20–30 µg EE/3 mg DRSP, 150 µg LNG/30 µg EE	NR	**127** (OC: 53; Non-user: 74)	OC (25 ± 1) Non-user (25 ± 1)
John et al. (58)	2000	Germany	At the basal level, NO production and release was enhanced by oral contraceptive use, while upon stimulation, NO bioavailability remained unchanged in the participants’ group.	Effects of oral contraceptives on the vascular endothelium in premenopausal women	Case control	COC	0.035 mg EE/0.125 mg LNG; 0.03 mg EE/0.15 mg DG; 0.03 mg EE/0.075 mg GSD; 0.03 mg EE/0.05 mg LNG; 0.02 mg EE/0.15 mg DSG.	1 year	**16** (OC: 8; Non-user: 8)	OC (27 ± 2) Non-user (27 ± 2)
Nadai et al. (59)	2015	Brazil	There was no significant difference in BP associated with the use of combined oral contraceptives containing DRSP irrespective of the EE dose used	Effects of two contraceptives containing drospirenone on blood pressure in normotensive women: a randomized-controlled trial	RCT	COC	30 μg EE/3 mg DRSP, 20 μg EE/3 mg DRSP	6 months	**44** (OC: 44; Non-user: NR)	OC (24.7 ± 4.5) Non-user (NR)
Straznicky et al. (60)	1998	Australia	OCs significantly increased 24 h systolic and diastolic blood pressure levels, triglyceride levels, and insulin area under the curve in users when compared with non-users.	Effects of oral contraceptive use and dietary fat intake on blood pressure, cardiovascular reactivity, and glucose tolerance in normotensive women	RCT	COC	0.03 mgEE/0.15 mg LNG,0.05 mg EE/0.125 mg LNG,0.03 mg EE/0.05 mg LNG, 0.04 mg EE/0.075 mg LNG, 0.03 mg EE/0.125 mg LNG	>1 year	**32** (OC: 16; Non-user: 16)	OC (29.8 ± 7.8) Non-user (31.3 ± 7.7)
Franceschini et al. (61)	2013	Brazil	A COC containing LNG is associated with a more pronounced reduction in FMD and increased IMT of healthy women than a COC containing CMA and non-hormonal contraception.	Effects of combined oral contraceptives containing levonorgestrel or chlormadinone on the endothelium	RCT	COC	EE 30 μg/CMA 2 mg, EE 30 μg/LNG 150 μg	6 months	**64** (OC: 43; Non-user: 21)	OC (24.2 ± 6.1) Non-user (28.3 ± 3.7)
Zahra et al. (62)	2019	Iran	OCs significantly increase the plasma levels of homocysteine (HCY), TG, cholesterol (TC), and LDL-c among users when compared with non-users.	Effects of low-dose contraceptive pills on the risk factors of cardiovascular diseases among 15-35-year-old women	A retrospective cohort study	COC	30 µg EE/150 µg LNG.	>1 year	**100** (OC: 50; Non-user: 50)	OC (30.1 ± 3.7) Non-user (30.1 ± 4.1)
El-Haggar and Mostafa (63)	2015	Egypt	The uptake of combined oral contraceptive pills (COCPs) significantly lowered adiponectin concentration and significantly increased leptin and resisting levels and the atherogenic index when compared with other studied groups.	To evaluate the associated cardiovascular risk in Egyptian healthy consumers of different types of COCPs.	Cross-sectional study	COC	30 μg EE/150 μg LNG, 0.03 mg EE/0.075 mg GSD, 30 μg EE/3 mg DRSP	6 months	**120** (OC: 90; Non-user: 30)	OC (31.2 ± 2.7) Non-user (31.7 ± 1.8)
Fallah et al. (64)	2011	Iran	Low-dose COC uptake results in a significant decrease in serum adiponectin concentration and an increased atherogenic lipid profile by significantly increasing LDL levels.	Adiponectin, leptin, and lipid profile evaluation in oral contraceptive pill consumers	Cross-sectional study	COC	30 μg EE/150 μg LNG	>1 year	**100** (OC: 50; Nonuser: 50)	OC (31.7 ± 7.9) Nonuser (33.9 ± 6.3)
Winkler et al. (65)	2009	Germany	OC treatments significantly increased triglyceride and Apo AI levels and HDL levels, while LDL levels were reduced in OC users when compared with non-users.	The effects of two monophasic oral contraceptives containing 30 μμg of ethinyl estradiol and either 2 mg of chlormadinone acetate or 0.15 mg of desogestrel on lipids, hormones, and metabolic parameters	RCT	COC	EE 30 μg/CMA 2 mg, EE 30 μg/DSG 0.15 mg	6 months	**43** (OC: 43; Non-user: NR)	OC (27.2 ± 5) Non-user (NR)
Piltonen et al. (66)	2012	Finland	The use of oral, transdermal, and vaginal combined contraceptives (CCs) decreases androgenicity, worsens insulin sensitivity, and increases the level of markers of chronic inflammation at the same rates.	Effect of alternative administration routes of CCs on androgen secretion, chronic inflammation, glucose tolerance, and lipid profile	RCT	COC, Patch, vaginal ring.	EE 20 μg/DSG 0.15 mg, EE 20 μg/norelgestromin 150 μg, EE 15 μg/120 μg etonogestrel	2 months	**54** (OC: 18; Non-user: NR)	OC (23.5 ± 3.1) Non-user (NR)
Marcelo et al. (67)	2014	Brazil	There were no significant alterations in blood pressure, heart rate variability, and baroreflex sensitivity of healthy women during a 6-month period of use of a COC containing EE and DRSP.	To evaluate the effect of a contraceptive containing 20 μg of ethinyl estradiol and 3 mg of drospirenone on the heart rate variability, baroreflex sensitivity, and blood pressure of healthy women.	Prospective clinical trial	COC	20 μg EE/3 mg DRSP	6 months	**69** (OC: 36; Non-user: 33)	OC (28.8 ± 1.1) Non-user (30.3 ± 1)

NR, not reported; COC, combined oral contraceptives; EE, ethinylestradiol; DSG, desogestrel; DNG, dienogest; DRSP, drospirenone; CMA, chlormadinone acetate; LNG, levonorgestrel; MPA, medroxyprogesterone acetate; GSD, gestodene; IGF-1, insulin-like growth factor 1; RCT, randomized control trial.

### 3.2. Quality assessment and risk of bias of the included studies

**Figure 2:**
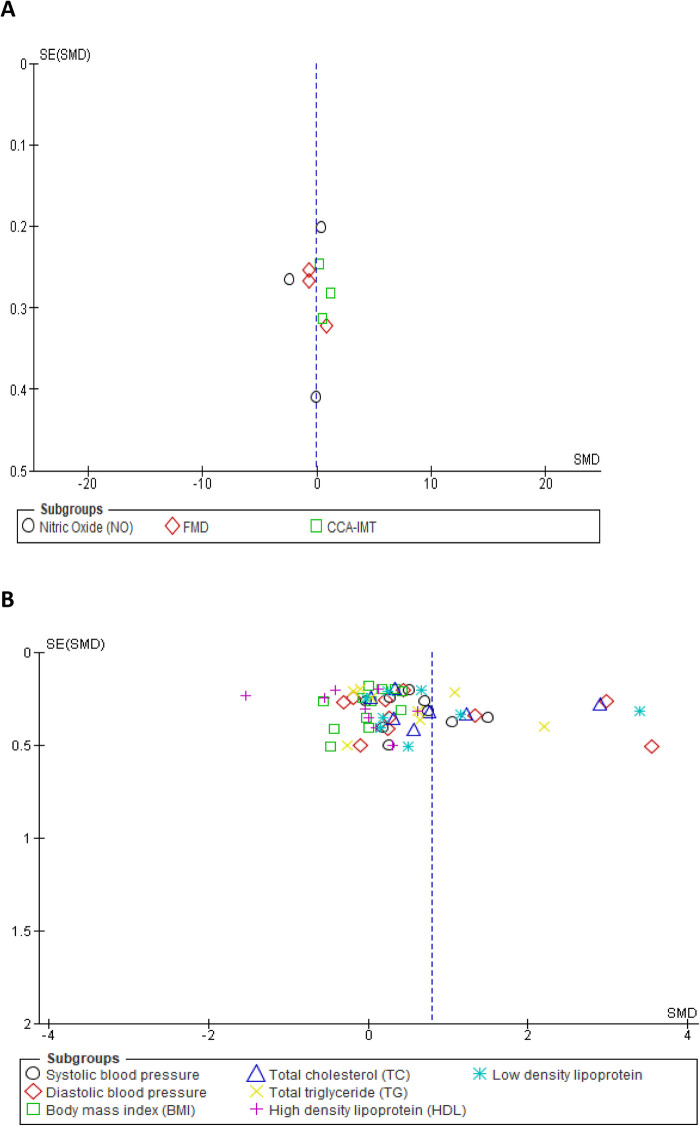
Quality assessment of the included studies.

The risk of bias was independently assessed by two reviewers (OAF and PVD) using the modified Downs and Black checklist (39). Overall, the included studies were rated as fair, with an average score of 18 out of a possible 26. Overall, the studies were scored as excellent for reporting the bias domain (with a score of nine out of a possible 10), poor for external validity (with a score of one out of a possible three), moderate for the internal validity domain (scoring three out of a possible seven), and moderate for selection bias (with a score three out of a possible six). The inter-rater reliability per domain was scored as *k* = 0.86 (CI = 0.8, 0.93) for reporting bias (perfect agreement), *k* = 0.54 (CI = 0.41, 0.68) for external validity (moderate agreement); *k* = 0.68 (CI = 0.53, 0.83) for internal validity (substantial agreement), and *k* = 0.63 (CI = 0.49, 0.77) for selection bias (substantial agreement) (Supplementary additional file S1, Figure 2).

### 3.3. The impact of OC use on reported markers of endothelial activation in premenopausal women

**Figure 3:**
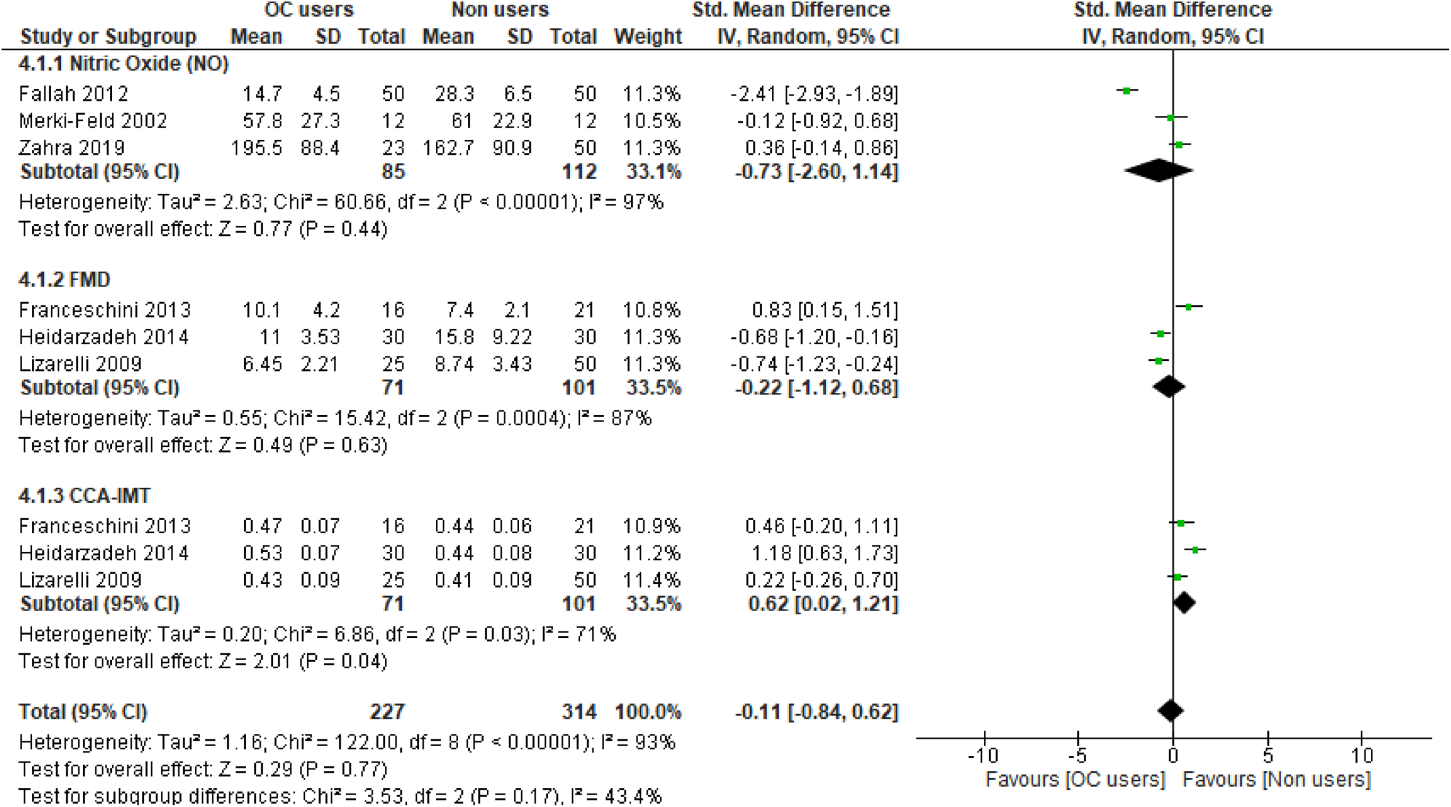
Forest plot of cellular and vascular markers of endothelial activation in premenopausal women on OCs vs. non-OC users.

Overall, the results of our meta-analysis showed little to no difference in the pooled estimate for endothelial activation among participants on OCs when compared with non-users [SMD = −0.11, 95% CI (−0.81, 0.60), *Z* = 0.30, *p* = 0.76, low certainty evidence]. However, these results showed a substantial level of statistical heterogeneity (*I*^2 ^= 94%, *p* < 0.00001) (Figure 3) and subgroup analyses based on study design, following which the reported measure of effect size of endothelial activation was estimated (Figure 3 and Table 2).

**Table 2:** Traditional cardiovascular-risk variables of included participants.

Effect Measure	Number of Studies	Number of participants	Effect estimate				
			Model	SMD	95% CI	*I*^2^, *p*-value	*p*-value
Blood pressure							
SBP	**12**, (46, 48, 62, 67, 49, 50, 55–58, 60, 61)	752	RE	1.96	0.94–2.97	97%, *p* < 0.001	3.78, *p* = 0.002
DBP	**12**, (46, 48, 62, 67, 49, 50, 55–58, 60, 61)	752	RE	1.74	0.71–2.78	97%, *p* < 0.001	3.3, *p* = 0.001
BMI	**14**, (46, 47, 61–63, 67, 48–50, 55–58, 60)	897	RE	0.22	−0.14–0.57	82%, *p* < 0.001	1.21, *p* = 0.23
Lipid metabolism							
Total cholesterol	**8**, (46, 48, 55, 56, 60, 62, 63)	536	RE	0.94	0.22–1.66	92%, *p* < 0.001	2.55, *p* = 0.01
HDL cholesterol	**9**, (46, 48, 49, 55, 56, 58, 60, 62, 63)	552	RE	−0.20	−0.64–0.25	82%, *p* < 0.001	0.85, *p* = 0.39
LDL cholesterol	**8**, (48, 55, 56, 58, 60, 62, 63)	509	RE	0.79	−0.04–1.59	92%, *p* < 0.001	1.93, *p* = 0.05
Triglycerides	**8**, (48, 55, 56, 58, 60, 62, 63)	528	RE	0.48	−0.02–0.99	85%, *p* < 0.001	1.87, *p* = 0.06
Glucose metabolism							
Fasting blood glucose	**3**, (55, 56, 60)	87	RE	0.07	−0.23–0.37	0%, *p* = 0.59	0.45, *p* = 0.65
Total effect estimate	**14**	4,320	−	0.74	0.47–1.01	94%, *p* < 0.001	5.41, *p* < 0.001

SBP, systolic blood pressure; DBP, diastolic blood pressure; HDL, high-density lipoprotein; LDL, low-density lipoprotein; RE, random effects, MD, mean difference; SMD, standard mean difference.

#### 3.3.1. NO level

The qualitative findings of our study, as reported in Table 1, showed that at the basal level, NO production and release was enhanced by OCs [second-generation (LNG) and third-generation gestodene and desogestrel (GSD, DSG) types], but upon stimulation with different dosages of acetylcholine, the plasma level of NO remained unchanged (58). Meanwhile, a study by Merki-Feld et al. showed that the use of second-generation (LNG) and third-generation (GSD) OC did not alter the plasma levels of nitric oxide (46). In contrast, the use of second-generation (LNG) OCs was associated with reduced plasma levels of NO when compared with the control group (55). However, the pooled estimate of our subgroup analysis suggests that OC use may result in little to no difference in theplasma level of NO when compared with non-OC users (SMD = −0.73, 95% CI (−2.60, 1.14), *p* = 0.44 (low certainty evidence) with a substantial level of heterogeneity (*I*^2 ^= 97%, *p* < 0.00001) (Figure 3).

#### 3.3.2. Flow-mediated dilation

The qualitative findings of our study, as reported in Table 1, showed that the use of fourth-generation drospirenone (DRSP) OC significantly increased flow-mediated dilation (FMD) in the brachial artery of the participants (43), which contrasted with the findings of other studies (49, 50, 61), where the use of second-generation levonorgestrel (LNG) and fourth-generation chlormadinone acetate (CMA) OC by the participants significantly lowered FMD when compared with non-users. However, the results of our meta-analysis suggest little to no difference in the pooled estimate for FMD in the participants on OCs when compared with non-OC users [SMD = −0.22, 95% CI (−1.12, 0.68), *p* = 0.63 (low certainty evidence) with a substantial level of heterogeneity (*I*^2 ^= 87%, *p* = 0.0004)] (Figure 3).

#### 3.3.3. Common carotid artery intima–media thickness

The qualitative findings of our study, as reported in Table 1, showed that the mean CCA-IMT was significantly higher in participants who used second- and third-generation OCs (50, 61), which contrasted with the findings of a study by Lizarelli et al. that reported no significant difference between users of the second-generation levonorgestrel (LNG) and non-users (49). However, the results of our meta-analysis showed a significant increase in the pooled estimate for CCA-IMT in participants not on OCs when compared with OC users [SMD = 0.62, 95% CI (0.02, 1.21), *p* = 0.04, low certainty evidence], although a substantial level of statistical heterogeneity was observed in these studies (*I*^2 ^=^ ^71%, *p* = 0.03) (Figure 3). Thus, our evidence suggests that OC use may result in a significant reduction in CCA-IMT among users.

### 3.4. Prevalence of traditional cardiovascular risk variables among OC users when compared with non-users

The overall pooled estimates of our meta-analysis suggest an increased CVD risk among OC users when compared with non-users [SMD = 0.73, 95% CI (0.46, 0.99), *Z* = 5.41, *p* < 0.001] (*I*^2^ = 94%, *p* < 0.001, low certainty evidence). However, due to a substantial level of heterogeneity, a subgroup analysis of the reported effect estimates was conducted (Table 2).

#### 3.4.1. Blood pressure measurements

##### 3.4.1.1. Systolic blood pressure

The qualitative findings of our study, as reported in Table 1, showed that systolic blood pressure increased significantly among users of second- (levonorgestrel; LNG) and third- (gestodene; GSD) generation COCs (56, 57, 60, 62, 64), which contrasted with those of a study by Franceschini et al. that reported a significant reduction among users of second (LNG)-generation COC when compared with non-users (61). However, several other studies reported a non-significant change in systolic blood pressure (SBP) among COC users despite the similarity in the duration of use (46, 48–50, 58, 67). Furthermore, the results of our subgroup analysis suggest a significant increase in the SBPof participants on OCs when compared with non-users [SMD = 1.96, 95% CI (0.94, 2.97), *p* = 0.002, low certainty evidence] and a substantial level of heterogeneity (*I*^2^ = 97%, *p* < 0.001) (Table 2).

##### 3.4.1.2. Diastolic blood pressure

The qualitative findings of our study, as reported in Table 1, showed that diastolic blood pressure (DBP) increased significantly among users of second- (levonorgestrel; LNG) and third- (gestodene; GSD) generation COCs (48, 57, 64), which contrasted with those of a study by Franceschini et al. that reported a significant reduction among users of second- (LNG) and fourth- (CMA) generation COCs when compared with non-users (61). However, several other studies reported a non-significant change among COC users despite the similarity in the duration of use (46, 49, 56, 58, 60, 62, 67). In addition, evidence from our meta-analysis suggests a significant increase in DBP of participants on OCs when compared with non-users [SMD = 1.74, 95% CI (0.86, 3.03), *p* = 0.001, low certainty evidence], although there was a substantial level of heterogeneity (*I*^2^ = 97%, *p* < 0.001) (Table 2).

#### 3.4.2. Body mass index

The qualitative findings of our study, as reported in Table 1, showed that the use of second- (levonorgestrel; LNG) and third- (gestodene; GSD, desogestrel; DSG) generation COCs does not significantly increase body mass index (BMI) (46, 47, 49, 50, 55–58, 60, 61–63, 67), which contrasted with those of a study by Asare et al. that reported a significant increase in BMI among users of the second- (LNG) generation COC despite the similarity in the duration of use (48). However, the pooled estimate of our subgroup analysis suggests that OC use may result in little to no difference in BMI when compared with non-users [SMD = 0.22, 95% CI (−0.14, 0.57), *p* = 0.23, low certainty evidence] and a substantial level of heterogeneity (*I*^2^ = 82%, *p* < 0.001) (Table 2).

#### 3.4.3. Lipid profile

##### 3.4.3.1. Total cholesterol

The qualitative findings of our study, as reported in Table 1, showed that second- (levonorgestrel; LNG), third- (gestodene; GSD), and fourth-generation (drospirenone; DRSP) COCs significantly increased the total cholesterol (TC) level among users when compared with non-users (48, 55, 62, 63). However, some studies reported no significant difference among users of second- and third-generation COCs when compared with non-users despite similarity in the duration of use (46, 49). Furthermore, evidence from our subgroup analysis suggests a significant increase in the total cholesterol level among OC users when compared with non-users [SMD = 0.94, 95% CI (0.22, 1.66), *p* = 0.01, low certainty evidence] and a substantial level of heterogeneity (*I*^2^ = 92%, *p* < 0.001) (Table 2).

##### 3.4.3.2. High-density lipoprotein

The qualitative findings of our study, as reported in Table 1, showed a significant increase in the high-density lipoprotein (HDL) level among users of second- (levonorgestrel; LNG) and third-generation (gestodene; GSD) COCs when compared with non-users (46, 56). However, these findings contrasted with the results of other studies that reported a significant decrease in the HDL level among users of second- (LNG) generation COC when compared with non-users and among third- (GSD) and fourth- (drospirenone; DRSP) generation COC users (49, 63). Nonetheless, several other studies reported non-significant changes in the HDL level among COC users despite similarity in the duration of use (55, 58, 60, 62). Furthermore, our subgroup analysis suggests that OC use may result in little to no difference in HDL levels when compared with non-users [SMD = −0.20, 95% CI (−0.64, 0.25), *p* = 0.39, low certainty evidence] and a substantial level of heterogeneity (*I*^2^ = 82%, *p* < 0.001) (Table 2).

##### 3.4.3.3. Low-density lipoprotein

The qualitative findings of our study, as reported in Table 1, showed an increased level of low-density lipoprotein (LDL) among users of second- (LNG) generation COC when compared with non-users and among third- (GSD) and fourth- (DRSP) generation COC users (48, 55, 62, 63). This contrasted with the findings of other studies that reported no significant differences among users of second- (LNG) and third- (GSD, DSG) generation COCs when compared with non-users despite similarity in the duration of use (55, 56, 58, 60). Nevertheless, the pooled estimate of our subgroup analysis suggests a significant increase in LDL levels among OC users when compared with non-users [SMD = 0.79, 95% CI (−0.04, 1.59), *p* = 0.05, low certainty evidence] and a substantial level of heterogeneity (*I*^2^ = 92%, *p* < 0.001) (Table 2).

##### 3.4.3.4. Triglyceride

The qualitative findings of our study, as reported in Table 1, showed an increased level of triglyceride (TG) among second-generation (LNG) users when compared with non-users (56, 62). While several studies reported no significant differences among users of second- (levonorgestrel; LNG) and third- (gestodene; GSD, desogestrel; DSG) generation COC users (48, 55, 58, 60), a study by El-Haggar and Mostafa showed a significant reduction in the levels of TG among users of second-generation COC (LNG) when compared with non-users and among third- (GSD) and fourth- (drospirenone; DRSP) generation COC users (63) despite similarity in the duration of use. In addition, the pooled estimate of our subgroup analysis suggests that OC use may result in little to no difference in triglyceride levels when compared with non-users [SMD = 0.48, 95% CI (−0.02, 0.99), *p* = 0.06, low certainty evidence] and a substantial level of heterogeneity (*I*^2^ = 85%, *p* < 0.001) (Table 2).

### 3.5. Glucose metabolism

#### 3.5.1. Fasting blood glucose

The qualitative findings of our study, as reported in Table 1, showed no significant change in fasting blood glucose (FBG) among users of second- (levonorgestrel; LNG) generation COC when compared with non-users (55, 56, 60). Moreover, the pooled estimate of our subgroup analysis also suggests that OC use may result in little to no difference in FBG levels [SMD = 0.07, 95% CI (−0.23, 0.37), *p* = 0.45, low certainty evidence] when compared with non-users (*I*^2^ = 0%, *p* = 0.59) and a low level of heterogeneity (Table 2).

### 3.6. Sensitivity analyses and publication bias

**Figure 4:**
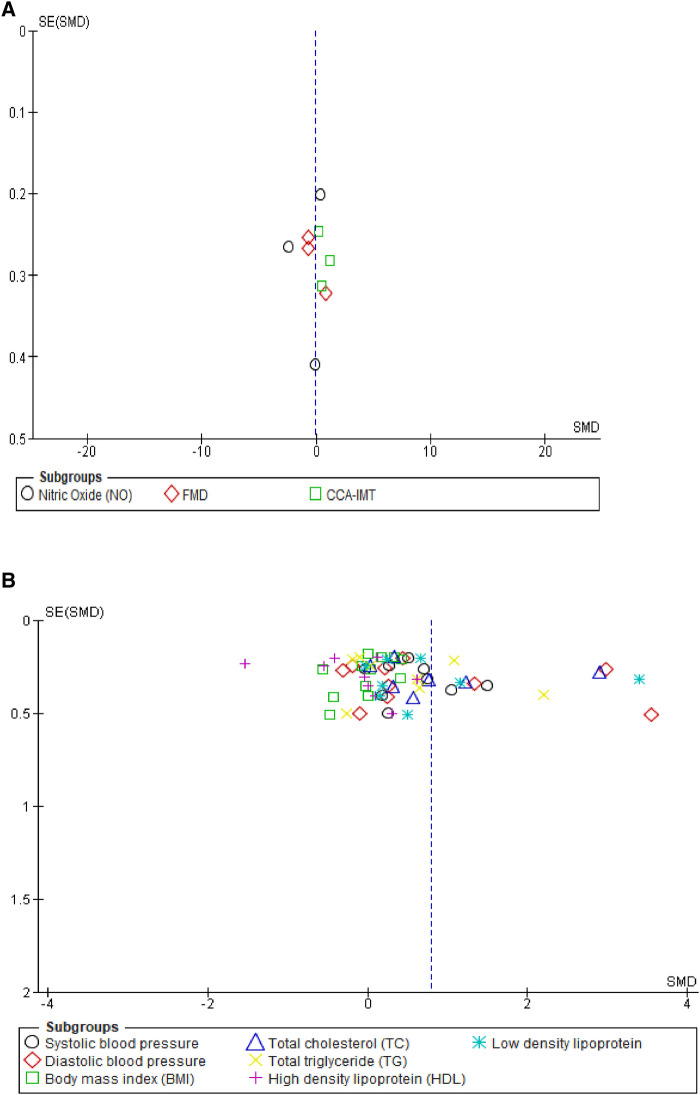
Funnel plot of vascular markers and cardiovascular risk factors showing a perfect symmetry. Hence, there was no publication bias in these studies. (**A**) Vascular markers, (**B**) Traditional cardiovascular risk variables.

We assessed the robustness of our results and further explored the sources of heterogeneity in the reported outcomes by performing sensitivity and subgroup analyses. The meta-analysis was repeated by a stepwise omission of studies based on the geographical location of each reported outcome. The sensitivity analysis of the traditional cardiovascular risk variables showed that studies conducted in Europe [SMD = 0.03, 95% CI (−0.21, 0.27), (*I*^2^ = 0%, *p* = 0.88)] and Australia [SMD = 0.33, 95% CI (0.05, 0.61), (*I*^2^ = 7%, *p* = 0.38)] had low levels of heterogeneity when compared with other studies conducted in Africa, Asia, and North and South America; however, the effect size was quite small in South America when compared with that in Africa, Asia, and North America (Supplementary additional file S1 and Table 3). This suggested geographical location to be a potential source of statistical heterogeneity in the included studies. However, an assessment of the funnel plot suggests evidence of publication bias (Supplementary additional file S1 and Figure 4B).

**Table 3:** Sensitivity analysis of outcomes based on geographical location.

Outcome	Geographical location	Number of studies	Omitted studies	SMD (95% CI)	*I*^2^ (%), *p*-value	Overall effect: Z, *p*-value
Vascular markers of endothelial dysfunction	All	**6,** (1–6)	None	−0.11 (−0.81, 0.60)	94%, *p* < 0.00001	*Z* = 0.30, *p* = 0.76
	Europe	**1,** (1)	**5,** (3–7)	−0.12 (−0.92, 0.68)	N/A	*Z* = 0.30, *p* = 0.76
	Asia	**3,** (3, 5, 7)	**3,** (1, 4, 6)	−0.39 (−1.83, 1.06)	97%, *p* < 0.00001	*Z* = 0.52, *p* = 0.60
	South America	**2,** (4, 6)	**4,** (1–3, 5)	0.17 (−0.49, 0.82)	82%, *p* = 0.0007	*Z* = 0.49, *p* = 0.62
Traditional cardiovascular risk variables	All	**14**, (1–6, 8–15)	None	0.74 (0.47, 1.01)	94%, *p* < 0.00001	*Z* = 5.34, *p* < 0.00001
	Europe	**2**, (1, 10)	**12,** (2, 3, 14, 15, 4–6, 8, 9, 11–13)	0.03 (−0.21, 0.27)	0%, *p* = 0.88	*Z* = 0.25, *p* = 0.88
	North America	**2,** (9, 14)	**12,** (1, 2, 13, 15, 3–6, 8, 10–12)	1.86 (−0.31, 4.04)	98%, *p* < 0.00001	*Z* = 1.68, *p* = 0.09
	South America	**4**, (4, 6, 11, 13)	**10**, (1–3, 5, 8–10, 12, 14, 15)	0.24 (0.01, 0.47)	72%, *p* < 0.00001	*Z* = 2.03, *p* = 0.04
	Africa	**2**, (8, 12)	**12**, (1, 2, 14, 15, 3–6, 9–11, 13)	1.44 (0.51, 2.38)	97%, *p* < 0.00001	*Z* = 3.02, *p* = 0.003
	Asia	**3**, (1, 2, 12–15, 3–6, 8–11)	**11,** (1, 2, 12–15, 3–6, 8–11)	1.30 (0.69, 1.91)	96%, *p* < 0.00001	*Z* = 4.19, *p* < 0.001
	Australia	**1**, (15)	**13**, (1, 2, 12–14, 3–6, 8–11)	0.33 (0.05, 0.61)	7%, *p* = 0.38	*Z* = 2.34, *p* = 0.02

**Table 4:** Summary of findings: use of oral contraceptives in premenopausal women compared with non-users.

Oral contraceptive treatment compared with non-users (controls)						
**Patient or population: [premenopausal women]**						
**Intervention**: [oral contraceptive]						
**Comparison**: [non-user]						
Outcomes	Anticipated absolute effects* (95% CI)		Relative effect (95% CI)	No. of participants (studies)	Certainty of the evidence (GRADE)	Comments
	Risk with [comparison]	Risk with [intervention]	–	197 (3 observational studies)	⨁⨁◯◯ Low^a,b^	
Cellular marker of endothelial activation—NO	–	SMD **0.73** **lower** (2.6 lower to 1.14 higher)				
Vascular marker of endothelial activation—FMD	–	SMD **0.22 lower** (1.12 lower to 0.68 higher)	–	172 (2 observational studies, 1 RCT)	⨁⨁◯◯ Low^a,b^	
Vascular marker of endothelial activation—CCA-IMT	–	SMD **0.62 higher** (0.02 higher to 1.21 higher)	–	172 (2 observational studies, 1 RCT)	⨁⨁◯◯ Low^a,b^	
Traditional cardiovascular risk variables	–	SMD **0.74 higher** (0.47 higher to 1.01 higher)	–	4,320 (12 observational studies, 2 RCTs)	⨁⨁◯◯ Low^a,b^	

CI, confidence interval; SMD, standardized mean difference.

*GRADE Working Group grades of evidence High certainty:* We are very confident that the true effect lies close to that of the estimate of the effect.

*Moderate certainty:* We are moderately confident in the effect estimate: the true effect is likely to be close to the estimate of the effect, but there is a possibility that it is substantially different.

*Low certainty:* Our confidence in the effect estimate is limited: the true effect may be substantially different from the estimate of the effect.

*Very low certainty:* We have very little confidence in the effect estimate: the true effect is likely to be substantially different from the estimate of the effect.

*The risk in the intervention group (and its 95% confidence interval) is based on the assumed risk in the comparison group and the relative effect of the intervention (and its 95% CI).

## 4. Discussion

The aim of this systematic review was to provide a comprehensive synthesis of the available evidence on the link between OC use and CVD risk in premenopausal women. Cumulative evidence summarized in this review highlights the impact of OC use on endothelial function and some traditional cardiovascular risk variables. The results of our study show that the use of progestin-only type of OC is associated with increased levels of plasma endothelin 1 (ET-1) in healthy young women (45). In contrast, the use of second-generation (levonorgestrel; LNG) and third-generation (gestodene; GSD) COCs does not significantly impact the plasma levels of ET-1 and NO (46). It is noteworthy that the imbalance in quotient between NO and ET-1 can impact the vascular tone. Meanwhile, a study by John et al. showed that the use of second-generation (LNG) OC significantly impacted the production and release of NO at the basal level and the levels of NO remained unchanged despite stimulating its release with acetylcholine and sodium nitroprusside (58). However, change in several hemodynamic, mechanical, and chemical factors, including blood pressure, vascular resistance, angiotensin II, as well as transforming growth factor-β, among others, can influence the activation and functions of endothelial cells leading to multiple inflammatory responses involving the innate and adaptive immune cells across the body system.

Furthermore, our study findings showed that fourth-generation (drospireneone; DRSP) OC significantly increased FMD (43). In contrast, the findings of other studies (49, 50, 61) involving the use of second-generation levonorgestrel (LNG) and another type of fourth-generation CMA OC showed lowered FMD. However, the reported pooled estimate of our meta-analysis showed no significant change in FMD in participants who used the second- (LNG) and third-generation (GSD, DSG) OCs (49, 50, 61).

More so, our study findings showed a significantly increased mean Common Carotid Artery Intima–Media thickness (CCA-IMT) in those who used second-generation (LNG) OC when compared with non-users and among fourth-generation (CMA) OC users (50, 61). However, the pooled estimate of our meta-analysis showed a significant decrease in CCA-IMT of participants on OCs when compared with non-users (49, 50, 61). In clinical settings, both FMD and IMT are strong predictors of endothelial dysfunction where FMD reflects early and predominant functional changes in the vascular wall, and IMT serves as a marker of more advanced structural changes (68). Nonetheless, understanding these changes may provide an insight into the power and effectiveness of the deep nerve stimulation to regulate systemic blood pressure (69).

Of note, endogenous estrogen is known to guard against vascular damage and atherosclerosis via the estrogen receptor (Eps), especially ERα and Erβ (70). However, the demonstrated changes in endothelial activation markers can be attributed to the type of progestin where a COC containing LNG was shown to result in 3–7.5-fold greater reduction in mean FMD among users when compared with non-users (61) and among users of fourth-generation (CMA) OC, which is derived from 17-hydroxyprogesterone, with high affinity for the progesterone receptor (PR) and moderate antiandrogenic activity (61). Furthermore, high androgenic properties associated with second-generation LNG progestin can antagonize the vasodilatory effects of estrogens and impact endothelial function (71, 72).

Furthermore, evidence emerging from our summary of findings showed that the OC use significantly increased systolic and diastolic blood pressure levels (60, 73, 74). Chronic use of COCs can induce increases in arterial pressure, primarily by activating the renin–angiotensin system (61) and via oxidative stress (75). However, some studies reported contradictory findings where the use of OCs did not significantly impact the blood pressure of the participants irrespective of the estrogen component (59, 67). Of note, endogenous female sex hormones are known to play a role in maintaining body fluid homeostasis (76) during the menstrual cycle. However, emerging evidence suggests that exogenous sex hormones may alter body fluid homeostasis in women of reproductive age (77, 78), which may depend on progestin type (76). While the progestin component may increase plasma volume through the combined mechanisms of increased osmolarity in the vascular space as well as overall expansion of ECF, the estrogen component may increase the plasma volume by reducing the operating point for osmoregulation of arginine vasopressin (AVP) and thirst, leading to a greater fluid retention in the vascular space (76).

AVP is a key hormone synthesized in the paraventricular and supraoptic nuclei of the hypothalamus (79, 80) they are released together with copeptin from the axonal terminals of the magnocellular neurons located in the posterior lobe of the pituitary gland (79). They are involved in the regulation of other body functions besides the control of the body's osmotic balance, respiratory and blood pressure regulation, sodium homeostasis, kidney functioning (80), fear conditioning, and love making (81–83). It is noteworthy that the synthetic progestins, apart from acting at the PR, can also influence the activity of other steroid receptors to induce androgenic, glucocorticoid, antiandrogenic, and antimineralocorticoid effects (84, 85).

Furthermore, findings from our data synthesis showed that the use of OCs is associated with dyslipidemia. Due to imbalance in the lipid profile, dyslipidemia may result in cardiovascular complications (86). The results showed that second- (LNG), third- (GSD), and fourth- generation (DRSP) COCs significantly increased the TC levels of OC users when compared with non-OC users (48, 55, 62, 63). In contrast, the findings from other studies showed that second- and third-generation COCs do not impact the TC level (46, 49). Furthermore, our study results showed that second- (LNG) generation COC increased the levels of LDL in users when compared with non-users, as also third- (GSD) and fourth- (DRSP) generation COCs (48, 55, 62, 63). This contrasted with the findings of other studies that showed that second- (LNG) and third- (GSD, DSG) generation COCs do not impact the LDL levels (55, 56, 58, 60). However, the pooled estimate of our meta-analysis showed that OC significantly increased the levels of TC and LDL in OC users when compared with non-users (62–67).

In addition, the results showed that second- (LNG) and third-generation (GSD) increased the HDL levels (46, 56). However, these findings contrasted with the results of other studies where second- (LNG) generation COC decreased the HDL levels when compared with third- (GSD) and fourth- (DRSP) generation COCs (49, 63). Nonetheless, the findings of several other studies showed that COCs do not impact the HDL levels (55, 58, 60, 62). More so, our study results showed that second- (LNG) generation COC increased the levels of TG (56, 62). On the other hand, second-generation COC (LNG) reduced the levels of TG when compared with the third- (GSD) and fourth- (DRSP) generation COCs (63). However, several other studies showed that COCs do not impact the TG levels (48, 55, 58, 60). Furthermore, the pooled estimate of our subgroup analysis showed an insignificant increase in the levels of TG and HDL among OC users.

Moreover, the results showed that COCs do not impact BMI (46, 47, 49, 50, 55–58, 60, 61–63, 67), although a study by Asare et al. showed that second- (LNG) generation COC increased BMI (48). However, the pooled estimate of our subgroup analysis showed that OCs do not impact BMI as well as FBG levels. Of note, emerging evidence showed the existence of regional disparities in cardiovascular disease incidence and mortality (87, 88). Moreover, three-quarter of the world's CVD deaths occur in low- and middle-income countries (89). Despite limited data on known risk factors to explain these regional variations in CVD among women of reproductive age, the results of our meta-analysis showed a high prevalence of traditional cardiovascular risk variables among OC users from North America when compared with Europe and other regions, which had the lowest prevalence.

There are several limitations in the evidence presented in this systematic review. These include substantial levels of statistical heterogeneity among included studies and unavailability of data on some prespecified effect measures. Therefore, caution should be exercised in interpreting and extrapolating these findings in different populations of various geographical locations.

## 5. Conclusion

The evidence presented in this review highlights the impact of second-generation (LNG) OC use on FMD, CCA-IMT, and NO levels in premenopausal women. In conclusion, evidence from our findings suggests that second-generation OC may result in little to no difference in endothelial activation. Although, among the variables assessed, our evidence suggests that the use of LNG may result in a significant reduction in CCA-IMT among users. Furthermore, our evidence suggests that the use of LNG may significantly increase other traditional cardiovascular risk variables. However, more independently conducted studies are needed to determine the long-term impact of individually available COCs on CVD risk.

## Data Availability

The original contributions presented in the study are included in the article/Supplementary Material, further inquiries can be directed to the corresponding author.
